# Endosymbiotic calcifying bacteria across sponge species and oceans

**DOI:** 10.1038/srep43674

**Published:** 2017-03-06

**Authors:** Leire Garate, Jan Sureda, Gemma Agell, Maria J. Uriz

**Affiliations:** 1 Centre d’Estudis Avançats de Blanes. Access Cala St Francesc, 14. 17300. Blanes (Girona) Spain

## Abstract

From an evolutionary point of view, sponges are ideal targets to study marine symbioses as they are the most ancient living metazoans and harbour highly diverse microbial communities. A recently discovered association between the sponge *Hemimycale columella* and an intracellular bacterium that generates large amounts of calcite spherules has prompted speculation on the possible role of intracellular bacteria in the evolution of the skeleton in early animals. To gain insight into this purportedly ancestral symbiosis, we investigated the presence of symbiotic bacteria in Mediterranean and Caribbean sponges. We found four new calcibacteria OTUs belonging to the SAR116 in two orders (Poecilosclerida and Clionaida) and three families of Demospongiae, two additional OTUs in cnidarians and one more in seawater (at 98.5% similarity). Using a calcibacteria targeted probe and CARD-FISH, we also found calcibacteria in Spirophorida and Suberitida and proved that the calcifying bacteria accumulated at the sponge periphery, forming a skeletal cortex, analogous to that of siliceous microscleres in other demosponges. Bacteria-mediated skeletonization is spread in a range of phylogenetically distant species and thus the purported implication of bacteria in skeleton formation and evolution of early animals gains relevance.

Symbiosis, whereby different biological species live together in intimate, long-term interactions, is regarded as a major source of evolutionary innovation^1^. Symbiotic associations of animals and microbes are widespread in marine ecosystems and play key ecological roles by contributing in an important, and often cryptic, way to the ecosystem biodiversity and stability. For example, shifts in symbiotic bacterial communities have been attributed to recurrent mass mortalities of corals and sponges^2^,^3^,^4^,^5^.

From an evolutionary point of view, sponges are ideal targets to study marine microbial symbioses, as they are the most ancient living metazoans on Earth and harbour highly diverse microbial communities^6^. The establishment of symbiotic relationships between sponges and prokaryotes has been traced back to the pre-Cambrian period^7^. However, despite the abundance of available sequences from sponge-associated bacteria and their long history, the evolutionary origins of these associations and the adaptive traits of the species involved are only beginning to be understood^8^,^9^,^10^,^11^.

Recently, a symbiotic association between the Atlanto-Mediterranean sponge *Hemimycale columella* (Bowerbank, 1874) and an unidentified calcifying bacterium, has been shown to produce thousands of calcite spherules^12^. The calcifying bacteria are contained within vacuoles in amoeboid, archaeocyte-like cells^13^, or ‘calcibacteriocytes’, where they divide by bipartition before becoming enclosed within a 100-nm thick calcite envelope. The calcifying bacterium is vertically transmitted to the sponge progeny^12^ via phagocytosis of maternal calcibacteriocytes as embryos grow^14^. Uriz *et al*.^12^ speculated that this type of eukaryote-prokaryote symbiosis might represent a relict mechanism involved in the evolution of skeletons in Lower Metazoa. Later, Blanquer *et al*.^15^ used pyrosequencing to retrieve a dominant alpha-proteobacterium in *H. columella* that represented up to 67% of the total sequences in the sponge and was similar to that obtained from the sponge *Cliona viridis* (Schmidt, 1862).

To gain insight into this newly identified symbiosis and its possible role in the evolution of skeletons in Lower Metazoa, we investigated whether the dominant alpha-proteobacteria reported by Blanquer *et al*.^15^ corresponded to the extremely abundant calcifying bacteria that form calcareous spherules in the sponge tissues^12^. We also explored the molecular diversity of the symbiotic calcibacteria, and their presence in other sponge species, to assess the occurrence of this symbiosis across sponges and oceans. Moreover, we explored whether the calcibacterium of *H. columella* is evenly distributed within the sponge tissues or accumulate in particular sponge zones, thus, fulfilling a potential skeletal function, and analyse the purported costs and benefits for the symbiotic partners.

To achieve these objectives, we performed ultrastructure studies using transmission electron microscopy (TEM) and scanning electron microscopy (SEM), and designed a molecular probe based on the dominant bacterial species of *H. columella*. We also conducted CARD-FISH experiments and cloned the 16 S rRNA gene of the calcibacterium from *H. columella* and *C. viridis* to examine their phylogenetic relationships.

## Results

### Light microscope observations

**Figure 1:**
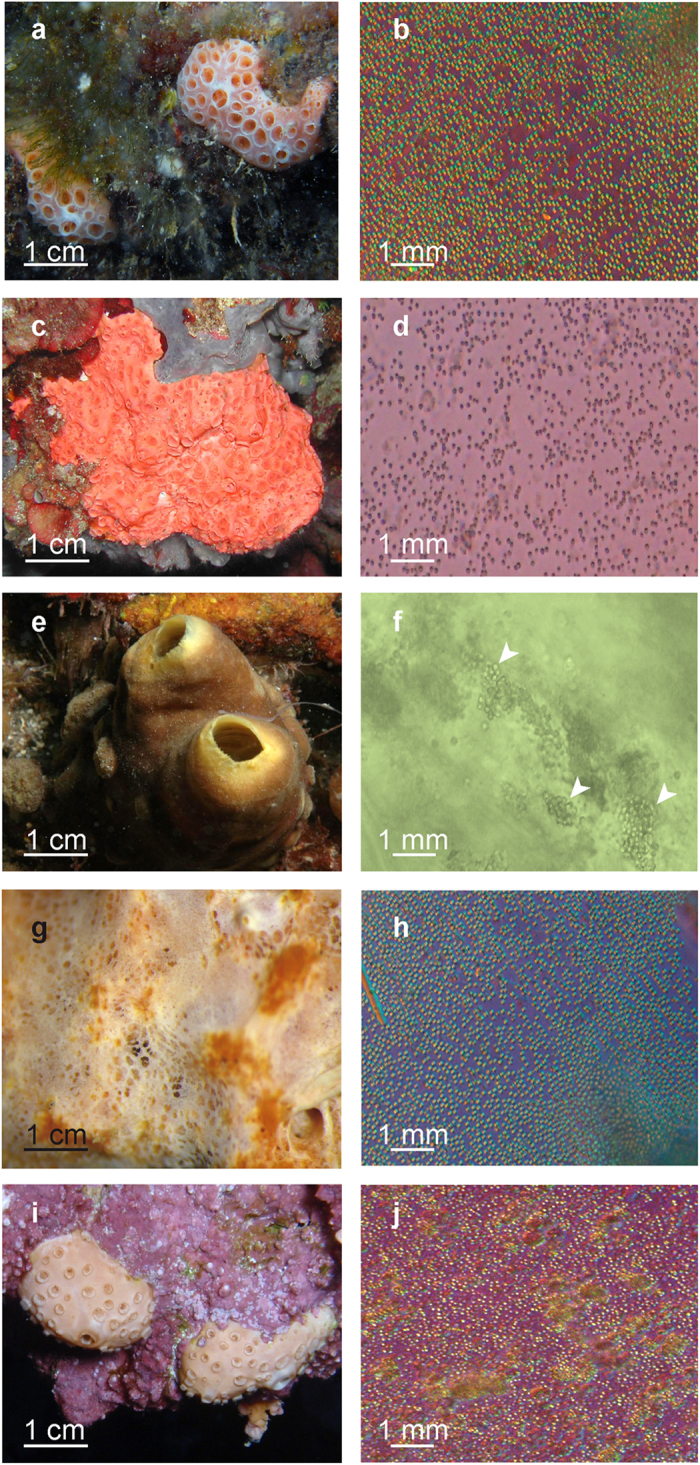
Living target sponge species and their calcareous spherules after fresh tissue squeeze. (**a**,**b**) Whitish morph of *Hemimycale columella*. (**c,d**) Pinkish morph of *H. columella*. (**e,f**) *Cliona viridis*. (**g,h**) *Prosuberites* sp. (**i,j**) *Crella cyathophora*.

Morphologically similar calcareous spherules, 1 μm in size, were observed in the sponges *Hemimycale columella, Cliona viridis, Prosuberites* sp., *Crella cyathophora* (Carter, 1869) (Fig. 1), and *Cinachyrella alloclada* (Uliczka, 1929) (not shown). Profuse numbers of spherules were released from the squeezed tissues of all the sponge species examined, except in *C. viridis* and *C. alloclada*, where they were less abundant.

**Figure 2:**
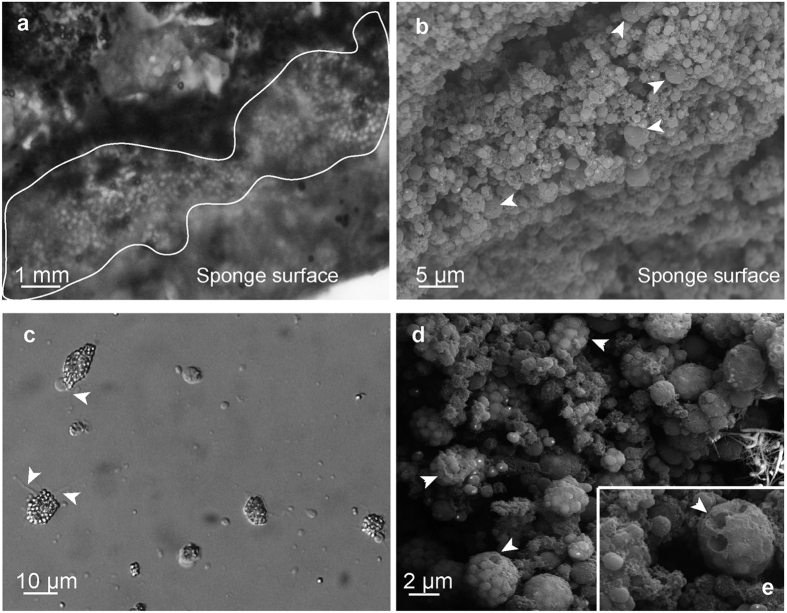
Calcibacteria and calcibacteriocytes from *Hemimycale columella* observed using light and SE microscopes. (**a**) Light microscope and (**b**) SEM pictures of calcareous spherules accumulated at the sponge periphery forming a kind of cortex. A few sponge non-granulose cells (arrowheads) are shown (**b**) among the dense layer of calcareous spherules. (**c**) Calcibacteriocytes showing pseudopodia and phyllopodia (arrowheads) while creeping across a solid surface (light microscopy). (**d**) Calcibacteriocytes full of calcified calcibacteria. (**e**) Close view of calcibacteriocytes (SEM). Hemispherical holes (arrowheads) correspond to the space previously occupied by calcibacteria.

The calcareous spherules were concentrated at the periphery of whitish individuals of *H. columella* (Fig. 2a,b). Moving sponge cells (calcibacteriocytes) full of calcified calcibacteria were recorded immediately after sponge disaggregation (Fig. 2c), which confirmed the amoeboid-like properties of these cells and their capacity to transport calcibacteria across the sponge tissues.

### Ultrastructure

**Figure 3:**
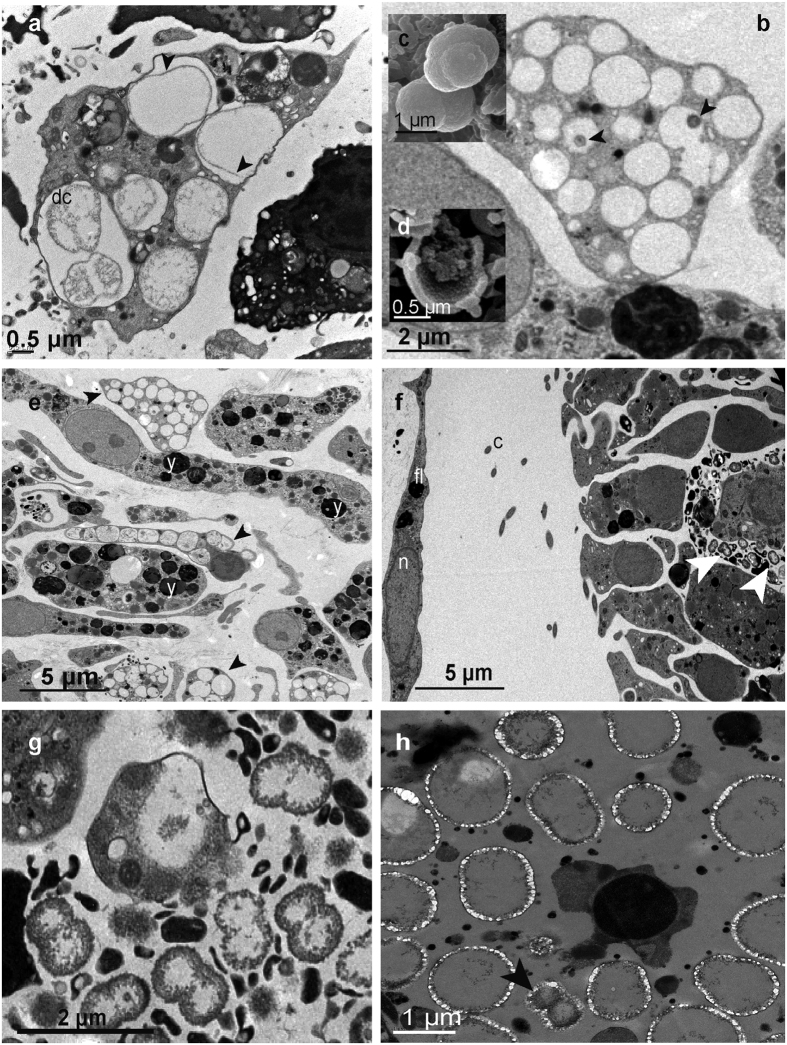
Ultrastructure images of *Hemimycale columella* tissues from adults and larvae. (**a**) Calcibacteria in adult tissue while dividing (dc) within a calcibacteriocyte (TEM). (**b**) Calcibacteriocyte showing the empty vacuoles previously occupied by calcified calcibacteria: arrowheads point to condensed bacterial remains (TEM). (**c**) SEM images of calcified calcibacteria undergoing division. (**d**) SEM image of a broken calcified calcibacterium showing a 100 nm thick calcareous crust and reduced organic matter inside. (**e**) Larval inner cells with yolk reserves (y) and larval calcibacteriocytes (arrowheads) full of remains of calcified calcibacteria (shown in the image as light to electron vacuoles with scarce organic material) (TEM). (**f**) Section of a larva within the follicle showing a calcibacteriocyte of maternal origin surrounded by non-calcified calcibacteria (arrowheads) released into the larval mesohyl: fl, follicular cell; (**c**) cilia of the larval peripheral cells; n, nucleus (TEM). (**g**) Free calcibacteria within the larva mesohyl showing profuse cell division by bipartition (TEM). (**h**) Pictures of free calcibacteria undergoing calcification in the sponge mesohyl (TEM). Calcification in the form of nanospherules can be observed. Arrowhead points to a calcibacterium enclosed within the calcareous crust while dividing.

SEM images of *H. columella* (whitish morph) showed huge numbers of calcareous spherules that had been released into the sponge mesohyl (Fig. 2b), as well as abundant, 10–15 μm calcibacteria-full calcibacteriocytes (Fig. 2d). The calcareous spherules were accumulated at the sponge periphery (Fig. 2b) forming a 2–3 mm thick layer, which concurred with light microscope observations (Fig. 2a). Some calcibacteriocytes showed hemispherical, ca. 1 μm in diameter holes, which corresponded to the space previously occupied by released calcibacteria (Fig. 2d). There were frequent images of calcibacteria enclosed within the calcareous coat as they divided (Fig. 3c).

TEM pictures of calcibacteriocytes showed abundant cellular vacuoles containing single or dividing calcibacteria (Fig. 3a). Most vacuoles contained a single bacterium, but several bacteria were also observed enclosed within a common calcareous coat after successive incomplete divisions (Fig. 3c). Calcified calcibacteria degraded in most cases, as indicated by the scarce or absent organic content within the calcareous crusts, but also formed condensed bodies, ca. 200 nm in size, that might correspond to starved forms (Fig. 3b). Uncalcified calcibacteria were spherical and relatively small (ca. 0.2–0.8 μm in cross-section), showed a thin bacterial wall and were found abundantly in the mesohyl of sponge larvae, after being released from engulfed maternal cells (Fig. 3f). There, they divided profusely by bipartition (Fig. 3g). Archaeocyte-like embryo cells (newly differentiated calcibacteriocytes) contained calcified calcibacteria (Fig. 3e). The calcareous crust that surrounded calcibacteria was clearly visible in SEM images of samples that were fixed for just 2 h (see methods) (Fig. 3h).

### Calcibacteria-specific probe

Table 1 shows the best 18 nt. long probe that targeted the prevalent alpha-proteobacteria sequence in *H. columella* (CAL32), as well as the 5′–3′ helpers, and competitors.

The non-sense probe, which was used as a negative control, did not hybridize in the samples (images not shown), and no hybridization occurred when the specific probe was assayed in the species *Crambe crambe* (Schmidt, 1862) (negative sponge control, images not shown), which confirmed that the hybridization signal observed was not an artefact and corresponded to the target calcibacteria.

### Catalysed reporter deposition fluorescence *in-situ* hybridization (CARD-FISH)

**Figure 4:**
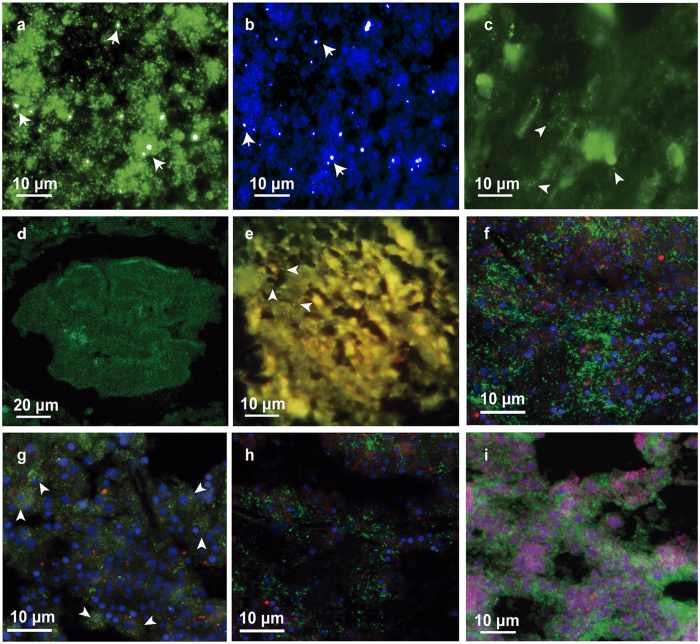
Study species hybridized with the CAL32L probe targeting the calcibacterium. (**a,b**) Filters containing isolated calcibacteria from *Hemimycale columella* (EM). (**c**) Filters containing isolated calcibacteria from *Cliona viridis* (EM). (**d**) *H. columella* choanosome harbouring larvae (CM). (**e**) Tissue section of *Cinachyrella alloclada* (EM). (**f**) Ectosome of *H. columella* whitish morph (CM); note that most calcibacteria are released into the sponge mesohyl forming dense aggregates. (**g**) Choanosome of *H. columella* whitish morph (CM); note that most calcibacteria are contained within calcibacteriocytes, surrounding the cell nucleus (arrowheads). (**h**) Ectosome section of *H. columella,* pinkish morph (CM). (**i**) Hybridized ectosome section of *Protosuberites* sp. (CM). Blue colour corresponds to sponge nuclei and bacteria nucleoid; green colour represents hybridization points; reddish colour results from self-fluorescence of cyanobacteria and pinkish colour results from overlapping DAPI blue-stained nuclei and reddish self-fluorescence of cyanobacteria. EM, Epifluorescence Microscopy; CM, Confocal Microscopy.

CARD-FISH using the designed probe (CAL32L), helpers, and competitors allowed us to detect the presence of the target *Ca.* Calcibacterium at high densities within the tissues of *H. columella,* (adults and larvae) and *Prosuberites* sp. and at lower densities in *C. alloclada* and *C. viridis* (Fig. 4). There were numerous hybridization points in the sponge mesohyl and within sponge cells (calcibacteriocytes) (Fig. 4g,h,i). Similarly, isolated calcibacteria from *H. columella* tissue on filters also hybridized in high numbers (Fig. 4a,b). This confirmed that the bacteria, which form the calcareous spherules within calcibacteriocytes, corresponded to the dominant alpha-proteobacteria sequence of *H. columella*.

The DAPI staining showed two discrete cell sizes, corresponding to DNA material from sponge cells and calcibacteria. Strong hybridization signals that overlapped with the majority of the DAPI-stained small points were observed with the CAL32L specific probe (Fig. 4a,b) in both tissue sections and extracted calcified calcibacteria, which proved the correspondence of the target bacteria with the calcareous spherules and the abundance of calcibacteria in the sponge tissues and cells. Negative controls, including the non-sense probe, a confirmed calcibacteria-free sponge, and a non-probe control yielded no hybridization signals confirming the precision of the hybridization and a lack of endogenous peroxidases in the sponge tissue. However, a few other non-hybridized bacteria were also DAPI stained in the sponge tissues. Moreover, although targeting cyanobacteria CARD-FISH was not performed, sporadic red/orange fluorescence was observed under green excitation (Fig. 4), indicating the occasional presence of photosynthetic bacteria in the sponge tissues.

**Figure 5:**
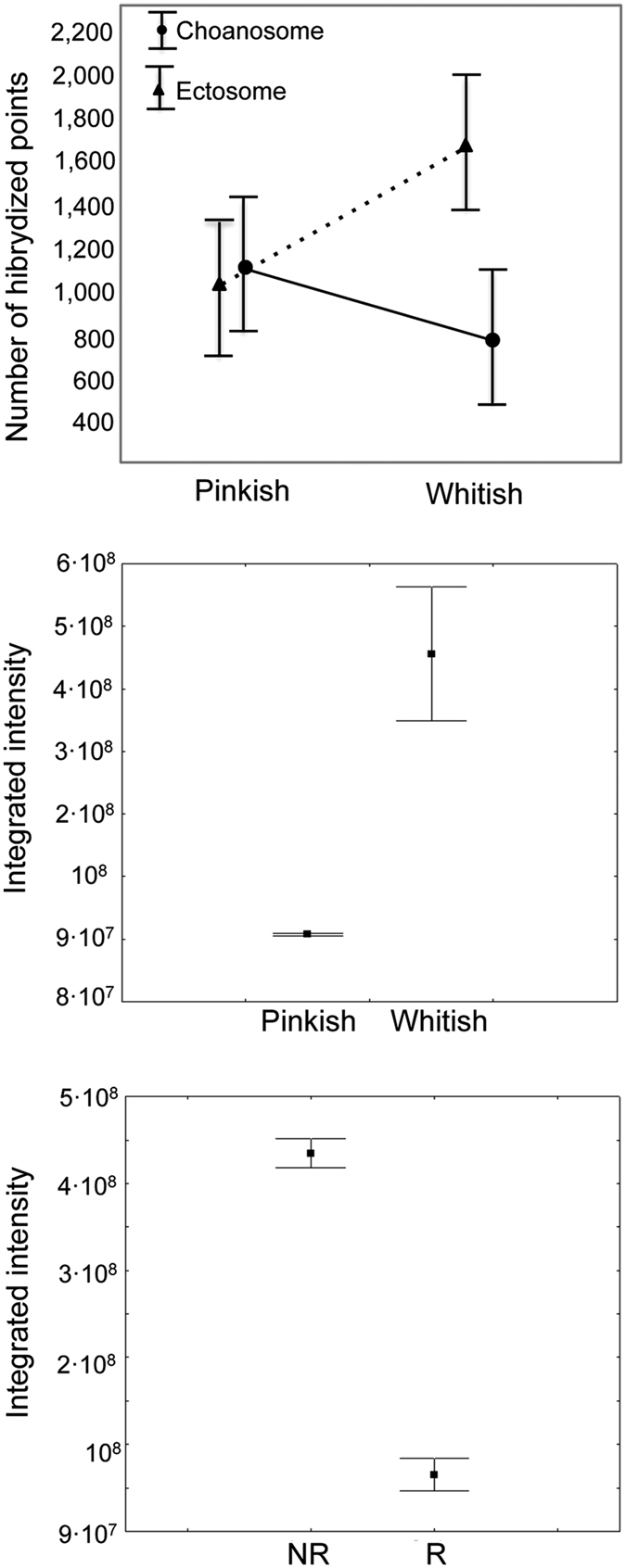
Average abundance of calcibacteria in *Hemimycale columella*. (**a**) Direct hybridization of calcibacteria extracted from the sponge tissue and quantified using epifluorescence microscopy. Vertical bars represented ± 95% confidence intervals. (**b**) Integrated intensity of the hybridization signal in the two colour morphs. Vertical bars represent ± standard errors. (**c**) Integrated intensity of the hybridization signal in two lifecycle stages (NR, non-reproductive; R, reproductive). Vertical bars represent ± standard errors.

Two-way ANOVA with colour morph and sponge zone as orthogonal factors (5 replicates per zone and colour morph) showed significant differences (N = 5, F = 12.14, p < 0.05) in the number of calcibacteria extracted from *H. columella* tissues and recorded through epifluorescence microscopy (EM) (Fig. 5a). However, the interaction between colour morph and sponge zone was also significant (p < 0.05), so that the abundance of calcibacteria in the two colour morphs depended on the sponge zone considered. T-tests on colour morph and sponge zone separately showed that whitish morphs had significantly higher numbers of calcibacteria (i.e. hybridized points) in the ectosomal region than pinkish morphs (t-value = −3.98, p < 0.001), whereas there were no differences in calcibacteria density between the choanosomes of both colour-morphs (t-value = 0.52, p = 0.67). Confocal microscopy of sponge sections confirmed the significant differences in calcibacteria density in the extracted spherules observed using EM (N = 3 each colour, Z = −1.96, p < 0.05, Mann-Whitney test) (Fig. 5b). Together, these results confirmed that calcified calcibacteria accumulated in large numbers at the sponge ectosome, conferring a whitish tinge to some individuals of *H. columella*, and that bacterial DNA remained within the calcareous spherules for an undetermined time after bacteria became calcified.

Larvae contained lower numbers of calcibacteria than adult tissue as non-reproductive individuals showed higher fluorescence values per tissue unit (Z = 7.32, p < 0.001, Mann-Whitney test) than sponges harbouring abundant larvae (Fig. 5c). The presence of larvae in reproductive individuals decreased the total integrated fluorescence of the sponge sections.

### Calcibacteria phylogeny

Cloning 16 S rRNA gene of the bacterial symbionts present in *H. columella* and *C. viridis* allowed us to recover two sequences, ca. 1,400 nt. long, that contained the 273 nt. calcibacterium fragment.

**Figure 6:**
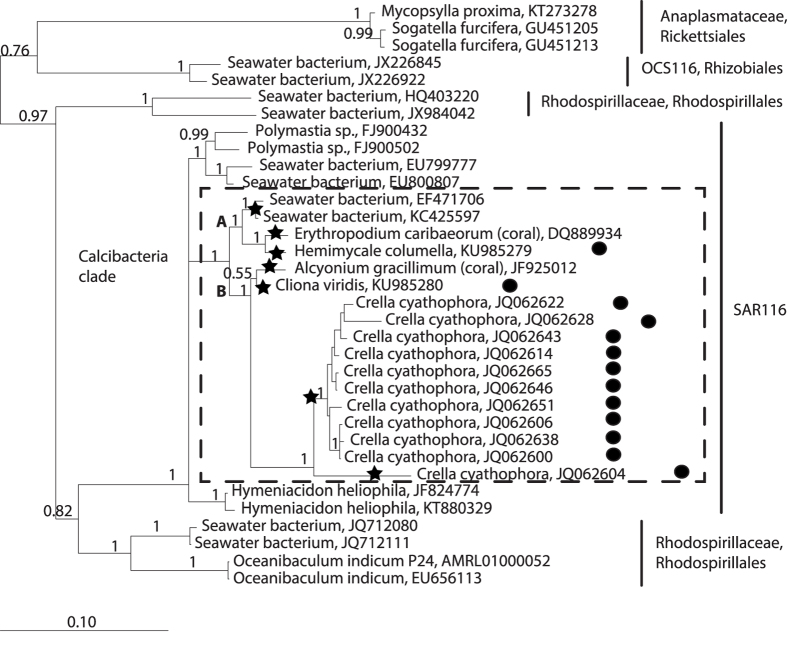
Bayesian phylogeny of the 16 S rRNA clones of calcibacteria from *Cliona viridis* and *Hemimycale columella,* and their closest sequences from the SILVA database. Sequence sources are included. The calcibacteria clade is shown within the dashed frame. Posterior probability values are indicated at each node. Bullets on the right indicate presence of ca. 1 μm calcareous spherules (the presence of spherules in cnidarians has not been explored). Seven calcibacteria OTU’s at >98.5% similarity (stars), are identified.

Bayesian phylogenetic reconstruction using the cloned sequences, plus their closest neighbouring sequences in the 16 S SILVA database and several outgroup sequences, retrieved a well-supported calcibacteria clade (1, posterior probability) that clustered bacteria from sponges harbouring calcareous spherules similar to those of *H. columella* along with sequences from two corals and two tropical seawater bacteria (Fig. 6). The calcibacteria clade contained 17 sequences, belonging to seven distinct calcibacteria OTUs with >98.5% intra-OTU similarity, which would correspond to seven calcibacteria species according to Kim *et al*.^16^. The calcifying bacterium of *C. cyathophora* differed from our cloned sequences of *C. viridis* and *H. columella*, in >5% and likely belonged to a different genus^17^.

According to the Bayesian phylogeny, the calcibacteria clade belonged to SAR116 (Alpha-proteobacteria), which appeared as a sister clade of some Rhodospirillales. It was split in two well supported subclades (1, posterior probability): subclade A contained the clone of *H. columella* (GenBank ID:KU985279), and that of the Alcyonacea coral *Erythropodium caribaeorum* (Duchassaing & Michelotti, 1860), and another clade with two bacterial sequences from tropical marine waters; subclade B clustered the *C. viridis* clone (GenBank ID: KU985280) with a clone from the Alcyonacea coral *Scleronephthya gracillimum* (Kükenthal, 1906) (previously *Alcyonium gracillimum*) from the North Pacific. This subclade was a sister clade of the *C. cyathophora* clone from the Indo-Pacific.

## Discussion

Ultrastructure images combined with molecular analyses have significantly improved our understanding of the symbiosis first discovered between *Hemimycale columella* sponges and calcibacteria. The specific probe designed enabled us to confirm the identity of the endosymbiont calcifying bacteria, which was surrounded by a calcareous crust, to quantify their abundance within the sponge tissues of *H. columella*, and to prove their accumulation at the sponge periphery, which suggest an exoskeleton function. A thin bacterial wall was made evident through TEM in healthy bacteria, which contradicts the lack of bacterial wall suggested by Uriz *et al*.^12^. The phylogenetic reconstruction of near-complete 16 S sequences from cloned *H. columella* and *Cliona viridis* classified the calcibacteria within the SAR116, close to one of the three Rhodospirillales clades^18^.

Altogether, the results from CARD-FISH and bacterial phylogeny indicate a temperate-warm geographical distribution of this symbiosis, which comprises at least seven bacterial OTUs and two potential genera, and appears to include demosponges and cnidarian hosts. The inclusion of two bacteria sequences from tropical waters within the calcibacteria clade suggests the presence of calcibacteria stocks in the water from which sponges and cnidarians might acquire them. Indeed, the symbiosis may be propagated by two mechanisms: vertical transmission from maternal tissues to the progeny, as evident in our study, and horizontal transmission from the environment, as suggested by the presence of free calcibacteria in seawater. Redundant mechanisms for assuring a relevant biological function are frequent in nature^19^ and two acquisition modes of symbiotic microbes have also been reported for other sponges^20^.

In marine environments, calcium carbonates and calcium phosphates are the most commonly precipitated minerals and have formed most invertebrate skeletons since the Cambrian explosion^21^. Particular metabolisms of both autotrophic and heterotrophic bacteria are known to induce mineralisation^22^,^23^,^24^,^25^,^26^,^27^,^28^,^29^. However, only a few cases of calcium precipitation mediated by endosymbiotic microorganisms have been reported so far in marine eukaryotes: calcification is facilitated by symbiotic microalgae (*Phaeocystis*) in the radiolarian *Acantharia*^30^, and by bacteria in the Foraminifera^31^.

Increased pH, which may result from bacterial metabolism, could promote calcium precipitation. Significant increases in pH have been recorded during the growth of *Escherichia coli*^32^, and an increased pH of at least one unit above seawater pH fostered calcification in Foraminifera vacuoles, even at high Mg2 + and low Ca2 + concentrations and low temperature^33^.

**Figure 7:**
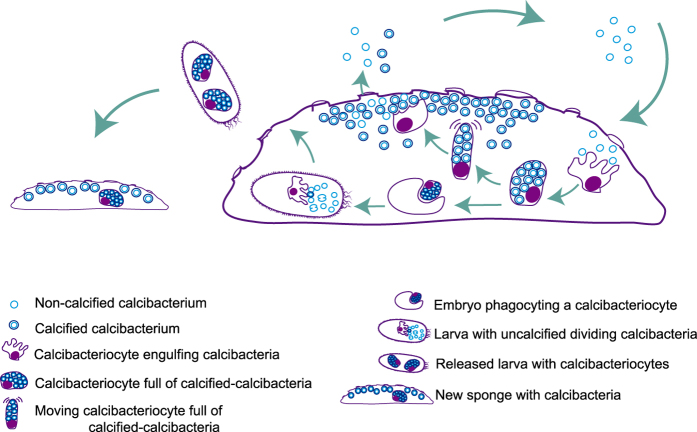
Proposed cycle of calcibacteria acquisition and vertical transmission to sponge settlers. Uncalcified calcibacteria enter the sponge with the inhaled water, are engulfed by the sponge ameboid archeocyte-like cells (calcibacteriocytes), which place them in cell vacuoles where bacteria calcification follows. Calcibacteria-full calcibacteriocytes move to the sponge periphery, where they disintegrate releasing the calcified calcibacteria, which accumulate forming a cortical calcareous layer. Embryos engulf maternal calcibacteriocytes during the maturation process. Maternal calcibacteriocytes disintegrate, releasing the calcibacteria to the larva mesohyl, where the extracellular pH conditions would prevent their calcification. Uncalcified calcibacteria divide in the larval mesohyl until they are captured by larval calcibacteriocytes where they calcified. Free larvae carry out calcibacteriocytes, and the cycle resume after larval settlement.

In our target symbiosis, calcification occurred within sponge cell vacuoles. We propose that the vacuole microenvironment changed over the course of bacterial growth, as nutrients are removed from the medium and bacteria expel waste products into the medium (Fig. 7). As a consequence, the pH of the vacuole may increase, and calcium carbonate nucleation and precipitation on the bacteria membrane is biologically induced^34^. A similar process of calcification has been observed in experimental studies of *Chromohalobacte*r *marismortui*^28^.

In general, sponge skeletons are either siliceous or calcareous^35^. Only a few relict sponges (the sclerosponges), which formed sponge reefs during the late Palaeozoic and Mesozoic eras^36^, have a double mineralisation system. Some parallels can be drawn between sclerosponges and the sponge species in this study. Both have siliceous spicules and a complementary calcareous skeleton layer. In both cases, spherules (spherulites in sclerosponges) are produced within the vacuoles of sponge cells (sclerocytes in sclerosponges or calcibacteriocytes in sponges in this study), which are then excreted and accumulate at the sponge periphery. However, some differences between the calcareous bodies produced by the two sponge types should be noted. In the sclerosponge *Astrosclera willeyana* Lister, 1900, calcareous bodies are solid, and 5 μm in diameter, and become cemented forming a mass skeleton at the sponge base^37^, whereas those of the sponges in this study are hollow and 1 μm in size, and remain free forming a cortical layer analogous to the siliceous, microsclere-constructed cortex of some Astrophorida demosponges^35^.

Studies on the formation of intracellular spherulites in the sclerosponge *A. willeyana* also report parallel traits with the bacteria-mediated calcareous spherules. Jackson *et al*.^38^ proposed that sponge genes of bacterial origin promoted calcification. Later, Jackson and Wörheide^39^ suggested that sponge cells use the remains of intracellular bacteria as a framework on which to initiate calcification. In both cases, the bacterial wall may act as a nucleation centre for the precipitation of calcium carbonate. Calcification, resulting in either 5 μm spherulites or 1 μm spherules, appears to be caused by the particular conditions (e. g. host enzymes or increased pH) in the sponge vacuoles, as it occurs within cell vacuoles.

It has been reported that many symbiotic microorganisms do not grow unconstrained in hosts^40^. Conversely, it is generally accepted that for mutualistic symbioses to become evolutionaryly fixed, benefits at the species level should compensate for the costs to the associated partners^41^. The most obvious benefit to sponges from their association with calcifying bacteria is the ‘low cost’ construction of an exoskeleton, which may serve as structural purpose and deter potential sponge predators better than secondary metabolites^42^. Protection against an increase in predators has been proposed as an evolutionary driver of exoskeletons in ancient animals during the Cambrian explosion^43^. By assuming that calcium precipitation around the bacteria is spontaneously triggered by increases in pH within the vacuole, the only cost of formation of the sponge exoskeleton would be the transport of calcified calcibacteria to the sponge peripheral zone.

However, the sponge mechanism of particle capture and transport may have not evolved primarily for bacteria. Calcibacteriocytes do not differ significantly from archaeocytes, which are moving cells that are genetically programmed to remove debris and undesired substances from the sponge mesohyl^14^. Archaeocyte-like cells pack bioactive metabolites in the form of spherules to prevent sponge self-toxicity^44^,^45^. These spherule-containing cells, or spherulous cells from their ultrastructural aspects^46^, have been observed to migrate to the sponge surface where toxins are released to the sponge boundaries to function in deterrent and/or allelochemical roles^44^.

In contrast, the benefits for calcibacteria are more difficult to ascertain. It has been experimentally demonstrated that some symbiotic microorganisms have an increased reproductive capacity and higher fitness within hosts relative to non-host environments^47^. Sponge tissues might offer protection from pathogens and predators, which are abundant in non-host environments^40^, and buffer nutrient ocean fluctuations that prevent the steady growth of bacteria over long periods^48^. However, considering the detrimental consequence of calcification for the bacteria, calcibacteria may also be more akin to ‘prisoners’ or ‘farmed crops’ than equal partners as in other bacteria-invertebrate symbioses^40^. Benefits might therefore be related to the propagation of the species. Symbiosis ensures that calcibacteria persist across sponge generations via the vertical transmission to sponge progeny. Moreover, the presence of free calcibacteria in seawater also suggests that viable calcibacteria are released back into the environment, which would allow the bacteria to form a species reservoir to facilitate dispersal and colonization of new invertebrate hosts.

Symbiotic relationships have been observed to be mostly stable over the lifetime of an individual host, from generation to generation, and over evolutionary time^49^. Thus, mechanisms must have evolved to correct potential deviations from the necessary holobiont homeostasis. In the calcibacteria-sponge association, the host should predominantly maintain proliferation of the dominant bacteria to a level compatible with host survival. Calcification appears to be the cost to bacteria for living in a more stable, predator-free, nutrient-rich environment. Once calcification prevents metabolic exchange between the bacteria and the vacuole medium, the calcibacteria may degrade or become starved. However, according to our TEM images, calcified calcibacteria recovered a steady growth phase as calcibacteriocytes broke and released purported resistant forms into the nutrient-rich mesohyl of sponge larvae. Then larval archaeocyte-like cells may engulf and transport them into cell vacuoles where calcification occurred (Fig. 7). This interpretation is based on *H. columella* observations but it can be safety extrapolated to other calcibacteria-bearing sponge species, as the cellular types involved in the process are similar in all of them (images not shown).

The current-day animal-bacteria symbioses, which likely existed when animals first appeared^50^, can provide key insights into Metazoa evolution. The reporting of bacteria-mediated calcification mechanisms in phylogenetically apart sponges suggests the implication of bacteria in the early evolution of the skeleton in the pre-Cambrian metazoans^51^. Although several molecular mechanisms are responsible for calcium precipitation and skeleton formation in Lower Metazoa^52^, those involving bacteria might be evolutionarily older and, thus, acquire new relevance in the light of these results. Paleogenomics approaches may help in the near future to confirm the presence of calcifying bacteria in early animals.

## Materials and Methods

### Sampling, sample preservation, and treatment

The sponge species studied all harboured 1 μm diameter calcareous spherules, similar to those reported in *Hemimycale columella*^12^ (Fig. 1). From one to three samples per species (depending on the species availability) were collected by SCUBA diving between 10 and 30 m depth in several seas: *H. columella* (whitish and pinkish morphs) and *Cliona viridis* from the Northwestern Mediterranean (Arenys de Mar, Spain); *Protosuberites* sp., and *Cinachyrella alloclada* from the Caribbean Sea (Florida, USA), and *Crella cyatophora* Carter from the Red Sea (Sharm el-Sheikh, Egypt).

Samples were preserved and treated according to the study purposes:

- For light microscopy, 50 mm^3^ samples were fixed in 4% paraformaldehyde, embedded in paraffin, and cut with an Autocat 2030 microtome (Reichert-Jung) to obtain 5 μm thick sections.
- For Scanning Electron Microscopy (SEM), ca. 4 mm^3^ sponge samples were fixed, critical point dried, and coated with gold-palladium^53^. Samples were observed using a Hitachi SEM at the Institute of Marine Sciences (ICM-CSIC).
- For Transmission Electron Microscopy (TEM), ca. 3 mm^3^ samples were fixed, rinsed with buffer, dehydrated, and embedded in a plastic resin^54^. Ultrathin sections were cut using an ultramicrotome (Ultracut Leica), and stained with uranyl acetate and lead citrate. Samples were observed using a JEOL 1010 TEM, implemented with Bioscan (Gatan) for image digitalization (Microscopy Unit of the Scientific and Technical Services of the University of Barcelona).
- For CARD-FISH experiments, ca. 50 mm^3^ samples were fixed in 4% paraformaldehyde for 4 h, then transferred to 70% ethanol, and embedded in paraffin. Histological sections were prepared according to the study needs (Table 1).
- For bacterial cloning, samples of *C. viridis* and *H. columella* were submersed in absolute ethanol immediately after collection and taken to the laboratory in a cooled container.

Different analyses were performed on different sub-sets of sponge species (see Supplementary Table S1).

### Calcibacterium location and quantification

We quantified the calcibacteria in the same set of *H. columella* individuals by two procedures to confirm that the calcibacteria were inside the calcareous spherules and to avoid possible biases in bacteria quantification in hybridized sponge sections due to signal overlapping in Epiflourescence Microscopy (EM):

- Hybridization of a filtered aliquot of the extracted calcibacteria (see procedure below) and observation using EM.
- Direct tissue hybridization and observation though Confocal Laser-Scanning Microscopy (CLSM).

### Calcibacteria extraction

Calcibacteria spherules were exhaustively extracted from fresh *H. columella* pieces. Pieces of ca. 1 g of fresh sponge (three individuals per colour morph and two regions per individual) were disaggregated and homogenized. Siliceous spicules were precipitated and then the pellet was discarded. The spicule-free homogenates were subjected to a series of centrifugations and re-suspensions^42^. One aliquot of the each final spherule suspensions (three per individual) was filtered through 0.2 μm pore filters and filters were CARD-FISH treated (Table 1).

### Oligonucleotide probe design

An oligonucleotide probe targeting the prevalent alpha-proteobacteria sequence in the sponge *H. columella* was designed using ARB software (http://www.arb-home.de/). The target sequence matched with two alpha-proteobacteria from the water column (GenBank ID: KC425597.1; GenBank ID: EF471706) and another from the cnidarian *Erytropodium caribaeorum* (GenBank ID:889934.1). The best probe was checked in silico with the online software MathFish (http://mathfish.cee.wisc.edu/) and its efficacy confirmed using the probe match tool in ARB. We also designed two ‘competitor sequences’ to avoid non-specific hybridizations, and two ‘helper sequences’ (Table 1) to hybridize the flanking regions of the specific probe. A non-sense probe (Non-EUB 338-I 5′-ACTCCTACGGGAGGCAGC-3′)^55^ was used as a negative control (Table 1). All the probes were synthesized using Biomers (http://www.biomers.net/).

### Catalyzed reporter deposition fluorescence *in-situ* hybridization (CARD-FISH)

We used CARD-FISH with the designed probe to verify whether the dominant sequence in the species *H. columella* corresponded to the abundant intracellular calcifying bacteria, observed using TEM and SEM, and to detect its presence in other sponge species harbouring 1 μm diameter calcareous spherules: *H. columella* (Poecilosclerida, Hymedesmiidae), *C. viridis* (Hadromerida, Clionaidae), *Protosuberites* sp. (Hadromerida, Suberitidae), and *C. alloclada* (Spirophorida, Tetillidae). CARD-FISH was also used to quantify calcibacteria within the main sponge regions (ectosome and choanosome) of whitish and pinkish morphs of *H. columella* (N = 3) and in two stages of the sponge life cycle: reproductive individuals incubating larvae and non-reproductive individuals.

Tissue samples that were fixed in 4% paraformaldehyde were dehydrated, embedded in paraffin, cut, deparaffined, and subjected to membrane permeabilization and inactivation of endogeneous peroxidases following procedures listed in Table 1 (modified from Pernthaler & Pernthaler^56^). Filters containing the extracted calcareous spherules were directly subjected to bacteria membrane permeabilization and inhibition of endogenous peroxidases. The optimum formamide concentration for the specific probe (i.e. 45%) was determined from assays at concentrations of 55%, 45%, and 35%. Following CARD-FISH, the sponge sections and the isolated spherules were DAPI stained to observe DNA. Dehydrated samples were mounted using Citifluor.

To discount self-fluorescence from the hybridized tissue, three sponge sections were treated according to the CARD-FISH protocol without adding the probe. Moreover, hybridization was assayed in tissues of the sponge *Crambe crambe*, a species that does not harbour the target calcibacteria^57^, to confirm that the hybridization signal observed was not an artefact.

### Quantification using epifluorescence microscopy

Hybridization was conducted on extracted calcibacteria from the sponge ectosome and choanosome of whitish and pinkish morphs. The resulting calcibacteria were re-suspended in sterilized seawater and an aliquot of this suspension was filtered through a 0.2 μm polycarbonate filter. Filters containing the calcibacteria were hybridized using CARD-FISH and observed using an epifluorescence microscope (EM) (Axioimager, Zeiss). Pictures were captured from 10 randomly selected fields at 100 × magnification using an Axiocam MR3 (Zeiss) digital camera (820 ms exposure time) attached to the microscope. We divided each field into 16 quadrats and counted the number of calcibacteria (hybridization points) in four randomly selected quadrats using Adobe Photoshop. The average number of calcibacteria per quadrat was multiplied by a factor related to the spherule concentration in the initial suspension.

### Quantification using confocal laser-scanning microscopy

We quantified calcibacteria in whitish and pinkish morphs of *H. columella* (N = 3) using a Leica TCS-SP5 confocal spectral microscope (Leica Microsystems Heidelberg GmbH, Universitat Autònoma of Barcelona) with a Plan-Apochromatic 63 ¥ 1.4 (oil HC ¥ PL APO lambda blue objective). A series of images (three fields per section) were taken every 1 μm (axis-z) across 6 μm thick histological sections to observe the emission signals of Alexa 488 and DAPI. The images were processed using Metamorph Imaging software (Universal Imaging Corporation, West Chester, PA, USA). We measured the integrated fluorescence intensity of the signal emitted by hybridized bacteria after removing the background fluorescence from the control samples.

### DNA extraction, amplification, and cloning

Samples of *C. viridis* and *H. columella* were submersed in absolute ethanol immediately after collection and taken to the laboratory in a cooled container. *H. columella* was extracted using Qiamp DNA stool kit (Qiagen) and *C. viridis* with DNeasy blood and tissue kit (Qiagen). One 16 S rRNA gene fragment, ca. 1,450 nt. in size, was amplified using universal primers 26 F and 1492R^58^. PCR conditions were as described previously^59^. PCR products were purified using QIAquick PCR Purification kit (Qiagen) and cloned using TOPO^®^ TA Cloning^®^ Kit for sequencing using One Shot^®^ TOP10 Chemically Competent *E. coli* (Invitrogen), according to the manufacturer instructions. Following colony growth, correct-size inserts were identified using PCR with T3–T7 primers, and purified and sequenced using the Sanger method (Macrogen Europe). Sequences containing the 273 nt. calcibacteria fragment^15^ were selected and aligned with the SILVA database using SINA web aligner. The alignment was merged into ARB software and improved using the Fast Aligner tool according to the secondary structure.

### Statistical analysis

Normality and homoscedasticity of data was verified^60^. Differences in calcibacteria abundance between sponge zones in different colour morphs were analysed using two-way ANOVA, with sponge colour (whitish or pinkish) and zone (ectosome or choanosome) as fixed orthogonal factors. Integrated fluorescence intensities obtained using CLSM were analysed using the Mann Whitney U-test, since data did not meet the assumptions of normality and/or homoscedasticity. Cloned sequences containing the 273 nt. calcibacterium fragment^5^, and the closest sequences were exported from ARB and used to construct a Bayesian phylogenetic tree using MrBayes 3.2 software. The GTR evolutionary model was used. Four Markov Chains were run with ten million generations sampled every 1000 generations. The chains converged significantly and the average standard deviation of split frequencies was less than 0.01 at the end of the run. Early tree generations were discarded by default (25%) until the probabilities reached a stable plateau (burn-in) and the remaining trees were used to generate a 50% majority-rule consensus tree.

The threshold used for considering a group of sequences belonging to the same “species” (now OTU) was a sequence similarity higher than 98.5% 16 while similarities >95% suggest same genus^17^.

## Additional Information

**How to cite this article**: Garate, L. *et al*. Endosymbiotic calcifying bacteria across sponge species and oceans. *Sci. Rep.* **7**, 43674; doi: 10.1038/srep43674 (2017).

**Publisher's note:** Springer Nature remains neutral with regard to jurisdictional claims in published maps and institutional affiliations.

## Supplementary Material

Supplementary Table S1

## Figures and Tables

**Table 1:** Different steps of the followed CARD-FISH protocol.

Step	Description
**1. Sample fixation**	1. Incubate sponge samples within 4% paraformaldehyde solution for 4 h2. Incubate samples in ethanol 70% during 18–24 h at 4 °C3. Keep samples in ethanol 70% at −20 °C
**2. Sample dehydration**	Sequential incubation of sponge samples in ethanol 96% and 100%, ethanol:toluene (1:1) 30 min each, and absolute toluene 15 min
**3. Embedding in paraffin and tissue sectioning**	1. Include samples in paraffin at 50 °C for 24–48 h2. Cut thick (6 μm) sections with an Autocut 2030 (Reichert-Jung) microtome and dry for 3 h at 40 °C
**4. Deparaffinization**	1. Incubate sections within Xylene for 10 min2. Rehydratation by sequential incubation in ethanol 100%, 96%, 70%, 10 min each3. Three baths in MiliQ water 5 min each, air dry
**5. Membrane permeabilization**	1. Incubate sections in 10 mg/ml Lysozime solution (Sigma USA), 0.05 M EDTA, 0.1 M Tris-HCl for 1 h at 37 °C2. Wash with MiliQ water for 2 min, air dry
**6. Endogenous peroxidases inactivation**	1. Incubate sections in 0.1 M HCl solution for 30–60 sec2. Wash with 1X PBS for 2 min3. Incubate in 3% H2O2 solution for 10 min4. Wash with MiliQ water and ethanol 96% 2 min each, air dry
**7. Hybridization**	1. Cover sections with hybridization buffer (1) solution together with the probe, helpers and competitors in a 3:100 volumetric ratio. Sequences (5′–3′) are:
	CAL32L: CCCCTCTATCTGCGGCGG
	Competitor I: CCCCTCTTTCTCCGGCGG
	Competitor II: CCCCTCATTCTGCGGCGG
	Helper 3′: YACAAGCTAATCGGACGCGGG
	Helper 5′: YACAAGCTAATCGGACGCGGG
	2. Incubate at 46 °C for 5 h with a solution of 45% formamide in humid chambers.
	3. Wash the sections with pre-warmed washing buffer (2) at 48 °C for 10 min
**8. CARD**	1. Wash sections in 1X PBS (pH = 8) for 15 min
	2. Cover sections with primary CARD substrate mix (3)
	3. Incubate at 46 °C for 20 min
	4. Wash twice in 1X PBS for 6 min at 46 °C and RT
	5. Wash twice in MiliQ water for 2 min each and air dry.

(1) The hybridization buffer was made by mixing 5 M NaCl, 1 M Tris-HCl (pH7.5), 20% sodium dodecyl sulfate (SDS), 10% (w/v) dextran sulfate, 10% Blocking Reagent and 45% formamide (Sigma). (2) Fresh whashing buffer was prepared by mixing 0.5 M EDTA, 1 M Tris-HCl, NaCl and 20% SDS in steryle miliQ water and warmed at 48 °C previously to the wash step. (3) The primary CARD substrate was made by mixing amplification buffer (10% (w/v) dextran sulfate, 2 M NaCl, 0.1% (w/v) blocking reagent, in 1X PBS (pH = 8)) with a freshly prepared H2O2 solution (0.15% in 1X PBS) at a ratio of 100:1. The needed volume of that primary CARD mix solution was mixed with 1 mg dye ml-1 tiramide-Alexa488 solution (Molecular Probes, Inc., Eugene, OR, USA) at a ratio of 500:5.
