# Supplementary material for: Endosymbiotic calcifying bacteria across sponge species and oceans
Source: Sci Rep. 2017 Mar 6;7:43674. doi: 10.1038/srep43674 (PMC5337934; doi:10.1038/srep43674)
Supplement: Supplementary Table S1 [file srep43674-s1.pdf]

Endosymbiotic calcifying bacteria across sponge species and oceans

Leire Garate, Jan Sureda, Gemma Agell, Maria J. Uriz*

Centre d'Estudis Avançats de Blanes. Access Cala St Francesc, 14. 17300. Blanes (Girona)

Spain

Species	Light microscopy	Electron microscopy	CARD-FISH	Calcibacteria quantification	Calcibacteria cloning
H. columella	X	X	X	X	X
C. viridis	X		X		X
Prosuberites sp.	X		X		
C. cyathophora	X				
C. alloclada			X		

Table 1. Analyses performed on the different sub-sets of sponge species
